# Influence of Global and Local Membrane Curvature on Mechanosensitive Ion Channels: A Finite Element Approach

**DOI:** 10.3390/membranes6010014

**Published:** 2016-02-05

**Authors:** Omid Bavi, Charles D. Cox, Manouchehr Vossoughi, Reza Naghdabadi, Yousef Jamali, Boris Martinac

**Affiliations:** 1 Institute for Nanoscience and Nanotechnology, Sharif University of Technology, 89694-14588 Tehran, Iran; Omidbavi@mehr.sharif.edu (O.B.); vosoughi@sharif.edu (M.V.); naghdabd@sharif.edu (R.N.); 2 Molecular Cardiology and Biophysics Division/Mechanosensory Biophysics Laboratory, Victor Chang Cardiac Research Institute, Darlinghurst, NSW 2010, Australia; O.Bavi@victorchang.edu.au (O.B.); C.Cox@victorchang.edu.au (C.D.C.); 3 Biochemical & Bioenvironmental Research Center (BBRC), 89694-14588 Tehran, Iran; 4 Department of Mechanical Engineering, Sharif University of Technology, 89694-14588 Tehran, Iran; 5 Department of Mathematics and Bioscience, Tarbiat Modares University, Jalal Ale Ahmad Highway, 14115-111 Tehran, Iran; Y.jamali@Modares.ac.ir; 6 Computational physical Sciences Research Laboratory, School of Nano-Science, Institute for Research in Fundamental Sciences (IPM), 19395-5531 Tehran, Iran; Y.Jamali@ipm.ir; 7 St Vincent’s Clinical School, Faculty of Medicine, University of New South Wales, Darlinghurst, NSW 2010, Australia

**Keywords:** membrane local curvature, mechanosensitive ion channel, continuum mechanics, local bending, finite element

## Abstract

Mechanosensitive (MS) channels are ubiquitous molecular force sensors that respond to a number of different mechanical stimuli including tensile, compressive and shear stress. MS channels are also proposed to be molecular curvature sensors gating in response to bending in their local environment. One of the main mechanisms to functionally study these channels is the patch clamp technique. However, the patch of membrane surveyed using this methodology is far from physiological. Here we use continuum mechanics to probe the question of how curvature, in a standard patch clamp experiment, at different length scales (global and local) affects a model MS channel. Firstly, to increase the accuracy of the Laplace’s equation in tension estimation in a patch membrane and to be able to more precisely describe the transient phenomena happening during patch clamping, we propose a modified Laplace’s equation. Most importantly, we unambiguously show that the global curvature of a patch, which is visible under the microscope during patch clamp experiments, is of negligible energetic consequence for activation of an MS channel in a model membrane. However, the local curvature (R_L_ < 50) and the direction of bending are able to cause considerable changes in the stress distribution through the thickness of the membrane. Not only does local bending, in the order of physiologically relevant curvatures, cause a substantial change in the pressure profile but it also significantly modifies the stress distribution in response to force application. Understanding these stress variations in regions of high local bending is essential for a complete understanding of the effects of curvature on MS channels.

## 1. Introduction

The generation and sensing of membrane curvature in biological systems is essential. Fully functioning cellular environments necessitate both the production and sensing of curvature over varying time scales to control a whole host of physiological processes [1]. At the molecular level the curvature may be generated by particular conical lipids (e.g., phosphatidylethanolamine, cardiolipin) or proteins interacting with or embedded within the biological membrane (e.g., Bar domain proteins). The sensing of this curvature is achieved via a number of mechanisms and an array of protein families (for review see [2]). One possible type of molecular curvature sensor are mechanosensitive (MS) ion channels. However, while numerous studies show that conical lipids or amphipathic compounds can modulate MS channel activity we still lack direct evidence that curvature local to a channel can *experimentally* induce channel gating [3,4,5,6,7].

MS channels can be defined as ‘being driven over all of their dynamic range by force alone’ [8]. This then distinguishes *bona fide* MS channels, such as Piezos, from those whose activity is simply modulated by force (e.g., Voltage-gated Na^+^ and K^+^ channels) but ultimately determined by other variables (e.g., membrane voltage, intracellular Ca^2+^ concentration) [9,10,11]. Whether these MS channels are gated directly via force applied from the surrounding lipids or they are tethered to structural scaffold proteins, it is patently clear that the mechanical state of the membrane is a crucial factor in their activation [12,13,14,15]. A widely used experimental paradigm to study MS channels is the patch clamp technique. However, the patch of membrane studied using this method does not represent a physiological scenario [16,17]. In fact, the mechanical environment of a patch of membrane ‘at rest’, in almost all cases, is under a high degree of tensional stress. In addition, the degree of stress is likely to be very different when we compare patch configurations, for example cell-attached patches compared to excised inside-out patches [18]. Despite a lack of full understanding of this molecular environment this methodology in combination with high-speed pressure clamps has provided a plethora of information about MS channel function [19]. One contentious and often confused point relates to how global patch curvature inside a patch pipette can potentially modulate channel activity (applied positive *vs* negative pressure) [20,21,22]. It is important to note that in a reconstituted bilayer system local curvatures are likely to be less prominent and important when compared to biological membranes containing structures such as caveolae. In cellular membranes local curvatures on the scale of those seen in caveolae can be expected to play a significant role in modulating MS channel function [23]. Although importantly no channel families have been robustly shown to localize to caveolae for example. In addition, many of these regions of high local curvature are associated with scaffold proteins and have linkages to the cytoskeleton and or extracellular matrix, which can modulate stress in the local region and contribute to signaling cascades [24].

**Figure 1:**
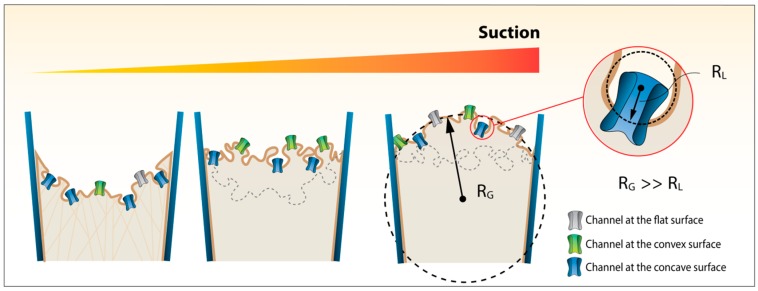
The difference between local and global curvature. Change in global (R_G_) and local (R_L_) radius of curvature of a membrane patch from a ‘resting state’ to an inflated dome state during applied negative pressure. The regions causing local curvature here in a biological membrane (e.g., caveolae) are of course not seen in a ‘pure’ lipid bilayer. As pressure is applied the regions of high local curvature flatten out. In a cellular environment this flattening of caveolae provides a degree of mechanoprotection by buffering the membrane tension in response to an applied force.

Here, we employ a finite element model to look at the effects that local and global curvature in a patch membrane can have on a model MS channel. We confirm that the global curvature of the patch (radius of curvature >0.1 μm) has little energetic consequence for a model MS channel. However, curvature at the local level (radius of curvature <100 nm) has a significant impact on the stress generated in response to membrane stretch (Figure 1 presents a schematic of global and local curvature in a patch). Moreover, the sensitivity of the channel to the applied force then dictates the degree with which local curvature impacts on its gating. In order to quantify this we introduce energetic terms widely used in material science related to the degree of change in curvature. In doing so we unequivocally show that the global curvature of a patch is of little consequence for a tension-sensing channel and rather the local curvature is what can potentially provide sufficient energy to modulate channel activity.

## 2. Materials and Methods

### 2.1 A More General and Accurate Approach for Tension Calculations

Here the generally unevenly curved membrane patch or part of it can be considered as an elastic or viscoelastic shell. In order to obtain more accurate results we make use of advanced and exact elasticity theories allowing more accurate analysis on the stress developed in response to the application of transverse loading resulting from mechanical force applied perpendicularly to the plane of the membrane patch (*i.e.*, mimicking negative pressure being applied to a patch pipette).

**Figure 2:**
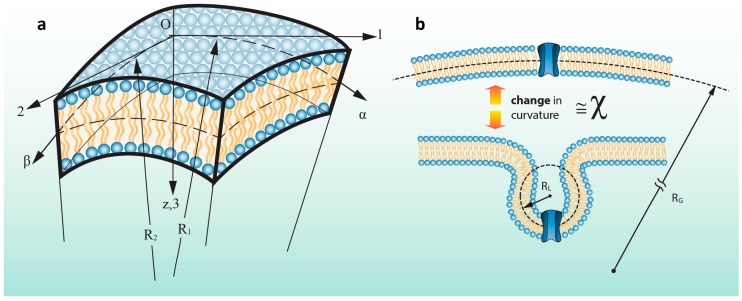
Schematic of membrane curvature. (**a**) An element of a membrane patch where the principal coordinates, Lamé parameters, radii of curvature, R1 and R2, change in curvature terms, $\chi_{1}$ and $\chi_{2}$ are shown. α and β are lines of curvature in the curvilinear coordinates; (**b**) Schematic description of the “change” in curvature term. This parameter, χ, is the change in curvature from the bilayer mid-plane between two different states.

Based on the elasticity equations, the radius of curvature of the patch in both the principle directions, 1 and 2, are named as $R_{1}$ and $R_{2}$, respectively (Figure 2a). And the in-plane deformations components of the patch subjected to the pipette pressure are named as u, v and the out of plane deformation is named as w. α and β are assumed to be the lines of curvature in the curvilinear coordinates. The changes in the curvatures of the patch in α and β coordinates are expressed as follows [25]:

(1-a) $$\chi_{1}=-\left[\frac{1}{A}\frac{\partial }{\partial \alpha }\left(\frac{u}{R_{1}}+\frac{1}{A}\frac{\partial w}{\partial \alpha }\right)+\frac{1}{AB}\frac{\partial A}{\partial \beta }\left(\frac{v}{R_{2}}+\frac{1}{B}\frac{\partial w}{\partial \beta }\right)\right]$$

(1-b) $$\chi_{2}=-\left[\frac{1}{B}\frac{\partial }{\partial \beta }\left(\frac{v}{R_{2}}+\frac{1}{B}\frac{\partial w}{\partial \beta }\right)+\frac{1}{AB}\frac{\partial B}{\partial \alpha }\left(\frac{u}{R_{1}}+\frac{1}{A}\frac{\partial w}{\partial \alpha }\right)\right]$$

where, *A* and *B* are Lamé parameters. They are quantities which relate a change in a curved length on the membrane surface to the corresponding change in the curvilinear coordinates, α and β. In fact, they can be inferred as distortion components transforming the change in curvilinear coordinates into the change in arc length of linear segments [25]. It should be considered that $\chi_{1}$ and $\chi_{2}$ are not curvature terms but changes in curvatures of the middle surface in the directions of the α and β due to the membrane patch bending (Figure 2b).

To obtain more precise results, large deflection theory of shells is adopted and adapted here to the special case of a spherical membrane patch. The strain components can be calculated as:

(2-a) $$\varepsilon_{1}^{z}=\varepsilon_{1}+z\chi_{1}$$

(2-b) $$\varepsilon_{2}^{z}=\varepsilon_{2}+z\chi_{2}$$

where $\varepsilon_{1}^{z}$ and $\varepsilon_{2}^{z}$ are the principal strains at an arbitrary point with distance *z* from the bilayer midplane. Taking into account the relationship between stress and strain, the stress distribution along the thickness of the membrane can be obtained as follows:

(3-a) $$\sigma_{1}^{z}=\frac{E}{1-\nu^{2}}\left[\varepsilon_{1}+\nu \varepsilon_{2}+z(\chi_{1}+\nu \chi_{2})\right]$$

(3-b) $$\sigma_{2}^{z}=\frac{E}{1-\nu^{2}}\left[\varepsilon_{2}+\nu \varepsilon_{1}+z(\chi_{2}+\nu \chi_{1})\right]$$

where E and ν are Young’s modulus and Poisson's ratio of the lipid membrane, respectively. Based on Equation (3), it is clear that the stress distribution varies through the membrane thickness and thus along the channel wall. The stress in each point of the membrane is a function of its distance from the midplane. Due to the cross section of a pipette and the spherical shape of the patch during patch clamp test, it can be assumed that $R_{1}=R_{2}=R$ and thus Equation (1) can be expressed in a simpler form. In this case, due to symmetry of applied pressure, curvature changes in both principal directions will be identical ($\chi_{1}=\chi_{2}$).

### 2.2. Finite Element (FE) Model

**Figure 3:**
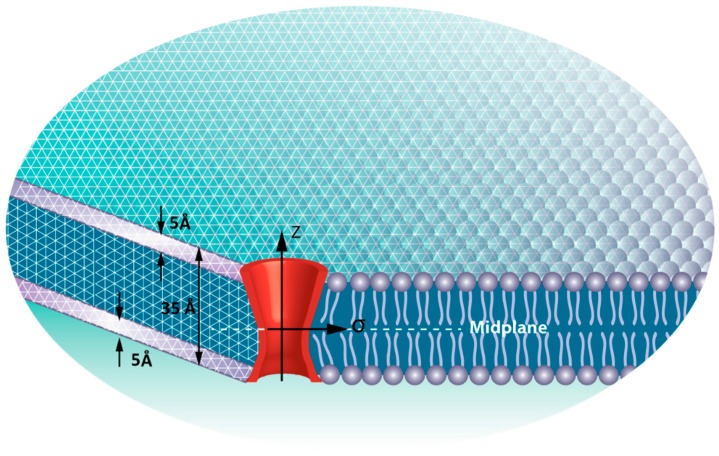
The FE model of the lipid bilayer and the incorporated MscL. The upper and lower layers represent the head groups and the core illustrates the tails of lipid bilayer. The thickness of the representative volume element (RVE) is 5 Å.

The stress variation was estimated using a finite element model employing ABAQUS commercial software (Abaqus/Standard; Simulia Dassault Systemes Simulia Corp. Providence, RI, USA). In this model, the lipid bilayer was considered as a three layer sandwich plate [26,27], in which the upper and lower plates represent the head groups and the core represents the tails of the lipid bilayer (Figure 3). This makes use of a mean field pressure profile described in Bavi *et al.*, 2014. The representative volume element (RVE) model of MscL used here was designed previously such that it is consistent with the MscL pore shape [28]. Therefore, the Young’s modulus and Poisson’s ratio of the RVE are assumed to be 40 MPa and 0.3, respectively. Our model also takes into account the hydrophobic pairwise interaction between the MscL RVE and the lipid bilayer. In this study, two membrane local curvatures of $C_{L}$ = 0.04 nm^−1^ and $C_{L}$ = 0.06 nm^−1^ were applied on the bilayer (spherical bending). These values correspond to the radius of curvature of *R = 1/*$C_{L}$ = 25 nm and *R = 1/*$C_{L}$ = 15 nm.

### 2.3. Patch Clamping

HEK293T cells were grown on 35 mm cell culture dishes (FluoroDish, World Precision Instruments, Inc, Sarasota, FL, USA) and transfected with an MscL-G22S-cGFP construct housed in a pTRE-tight vector using Lipofectamine 3000 (Invitrogen, Carlsbad, NM, USA) as per manufacturers protocol. Confocal images were made using a water immersion long working distance objective (×63; NA 1.15; Carl Zeiss, Oberkochen, Germany) on an inverted confocal microscope (LSM 700; Carl Zeiss, Oberkochen, Germany). Concomitant cell-attached recordings were carried out in a bathing solution consisting of 140 mM NaCl, 3 mM KCl, 1 mM CaCl_2_, 10 mM glucose and 10 mM HEPES (pH 7.2) adjusted using NaOH. The pipette solution contained 140 mM NaCl, 3 mM KCl, 1 mM CaCl_2_ and 10 mM HEPES (pH 7.2) again adjusted using NaOH. Negative pressure was applied to patch pipettes using a High Speed Pressure Clamp-1 (HSPC-1; ALA Scientific Instruments, Farmingdale, NY, USA). Borosilicate glass pipettes (Sigma, St. Louis, MO, USA) were pulled using a vertical pipette puller (PP-83, Narashige, Tokyo, Japan) to produce electrodes with a resistance of 2.5–3.5 MΩ. MscL-G22S-cGFP currents were amplified using an AxoPatch 200B amplifier (Axon Instruments, Union City, CA, USA), and data were acquired at a sampling rate of 10 kHz with 1 kHz filtration and analyzed using pCLAMP10 software (Axon Instruments, Union City, CA, USA). 

## 3. Results

### 3.1. Calculating Tension in a Membrane Patch

The pertinent variable for all currently known mechanosensitive channels gated according to the force from lipids concept is membrane, or more precisely, bilayer tension [10,29,30,31,32]. Estimating tension in a patch clamp experiment is not a trivial task and to date has been achieved using Laplace’s equation. Usually, a simplified form of the Laplace equation is employed (to see a more general form, please see the Supplementary Information):

(4) $$T=\frac{PR_{G}}{2}$$

where $R_{G}$ and T are the global radius of curvature and in-plane tension respectively. 

**Figure 4:**
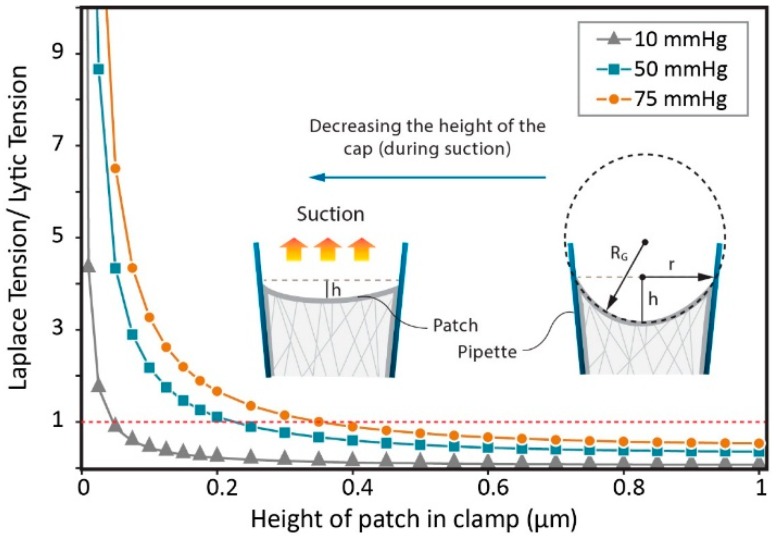
The effect of the flattening of the patch on the ratio of the membrane tension distribution and the lytic tension (calculated using the Laplace equation).

When negative pressure is applied to a cell-attached patch the shape changes from the resting state (usually downward curvature in a cell-attached patch) via a transient flat state to an inflated dome. In this scenario, it is patently clear that the stress, as well as the adhesion energy, get several times bigger than the realistic lytic tension of the membrane. Incidentally the membrane–glass adhesion energy, *T_a_*, can be estimated as [33];

(5) $$T_{a}=\frac{\sqrt{{R_{G}}^{2}-r^{2}}}{2}\times P$$

where *r* is the pipette radius, and *R_G_* is the radius of patch. The lytic tension of a membrane is of course dependent on its lipid constituents and can range from 7 mN/m to 20 mN/m for various ‘pure’ lipid compositions [5,34,35,36]. The lytic tension of biological membranes seems to be lower and the reason for this is in itself interesting. For example, we have previously shown that cholesterol alone can affect the lytic tension of bilayers and it is likely that the level of protein inclusions and lipid domains present in a cell membrane also vastly affect the lytic tension [5]. Here, we assume the average lytic tension of a membrane to be in the range of ~13 mN/m, thus in a patch clamp experiment with a 2 μm diameter pipette, applying −50 mmHg, while h = 0.05 μm (Figure 4), the estimated tension is ~4 times bigger than the lytic tension based on the Laplace Equation.

The adhesion energy presents one obstacle to accurately calculate tension in a membrane patch using the simplified form of Laplace’s equation. For example, MscL and other MS channels (e.g., MscS, Piezo1, TREK-1 and TRAAK) have been reported to occasionally exhibit spontaneous activity in patch clamp experiments [10,22]. In the case of MscL the frequency of such events increases by decreasing the thickness of the lipid bilayer [5,37]. A likely explanation for this it is that before applying any suction (resting state), the patch membrane is in a pre-tension state (because of capillary forces as well as the interaction between the membrane and the glass), *i.e.*, a resting level of tension. This tension may be of sufficient magnitude to lead to activation of channels especially those particularly sensitive to applied force such as some members of the MscS superfamily or Piezo channels. In the next step and by applying negative pressure (suction), the curved surface of a patch with a height of *h*, tends to expand in the pipette and so the area of the membrane changes from A = π(h^2^ + r^2^) to A = πr^2^ (see Figure 2), where r is the radius of the pipette in the junction of the patch and the pipette wall [33]. Since the flat state has a smaller area, the excess area wrinkles the patch and the tension is released. Thus, this may result in the rescue of apparently “spontaneous” channel activity and channel closure as, for example, in the case of mechano-gated K2P channels TREK-1 and TRAAK. In the third step (inflated dome state), with increasing suction, the patch reforms a spherical cap and channels reopen again.

Here, we aim to show that modification of Laplace’s equation can interpret the wrinkling due to the addition of the patch area during membrane flattening. In this case, because the radius of curvature of the patch is infinite in the flat state (from a mathematical point of view), based on the Equations (1) and (2), the distributed tension and adhesion energy are infinite as well. However, according to the experimental results, the channel activity during wrinkling [33], or patch buckling [38] is negligible and the current magnitude is almost equal to zero. 

We and others believe that global patch curvature is not important in channel activation exemplified by the fact that channels do not activate when the membrane wrinkles upon passing from inward to outward curvature [22,38]. However, given the experimental evidence related to amphipaths local curvature is much more likely to be a relevant modulatory stimulus. It is obvious that Laplace’s equation neither interprets nor is useful when considering regions of high local curvature. 

In the next section we discuss the drawback of Laplace’s equation to estimate tension in membrane areas characterized by high local curvature (such as microvilli and caveolae). Obviously such considerations are less important when looking at ‘pure’ bilayers containing reconstituted channels than when looking at biological membranes. In addition, the application of Laplace’s law in any form is not the most accurate way to calculate tension in a heterogeneous cellular membrane where more complex mechanical models are likely to perform much better [39]. However, Laplace does give us an upper limit on the tension generated and here we propose a mathematical formulation for the calculation of membrane tension induced by local membrane curvature. We then examine the stress distribution using finite element analysis and compare the computational results with experimental data.

### 3.2. Estimation of Stress in Regions of High Local Curvature

**Figure 5:**
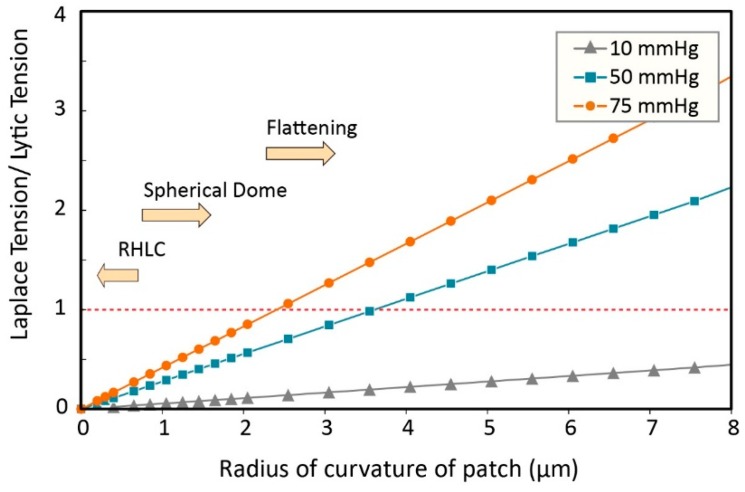
The effect of the radius of curvature on ratio of the distributed tension and the lytic tension. By decreasing the radius of curvature to the RHLC (Region of High Local Curvature), the calculated tension using the simplified form of the Laplace’s law approaches zero. This implies that all of the channels in RHLC don’t sense the membrane tension produced by the applied negative pressure.

There are many regions of high local curvature in cells; clathrin coated pits, microvilli, caveolae, cisternae of mitochondria, areas of the ER and Golgi membranes, to name just a few. If we take the radius of curvature seen in microvilli (0.05 μm [40]), this is about one-twentieth of a ‘usual’ patch radius. Thus according to the Laplace equation, the pressure required to activate an ion channel located in one of the microvilli should be 20 times the pressure required to activate the channel that is located in the membrane surface. In other words, according to the simplified form of the Laplace’s equation, the smaller the radius of microvilli or caveolae, the less distributed stress (Figure 5). 

For example if we take an adipocyte as an example they contain on average one million caveolae which can increase the surface area of the plasma membrane by 50% [41]. Consequently, Laplace’s equation in its rudimentary form is inadequate to determine the membrane tension in this case. As shown in the next section the Laplace equation can be modified so that it could be applied to all states that a membrane patch can assume during a patch clamp experiment. 

### 3.3. Modified Laplace’s Equation and Equivalent Membrane Patch Model

By proposing an equivalent radius of curvature, we can modify the Laplace equation providing a more adequate description of the behavior of a membrane patch in the pipette. The total tension in the patch can be separated into two terms: the resting tension (pre-stress) due to the initial resting forces (e.g., Cytoskeleton linkages, capillary forces between the pipette glass and the patch membrane), $T_{rest}$, and the tension generated by applying negative pressure (suction), $T_{Lap}$:

(6) $$T_{total}=T_{rest}+T_{Lap}$$

Thus, one may assume an equivalent curvature as:

(7) $$C_{equ}=C_{obs}+C_{rest}$$

where $C_{equ}$ is the equivalent curvature of the patch, and $C_{rest}$ is the curvature of the patch in the rest state; $C_{obs}$ is the apparent curvature which can be observed during the test. According to the inverse relation between the curvature and the radius of the curvature (C=1/R) and using Equation (7), one may obtain:

(8) $$R_{equ}=\frac{R_{rest}\times R_{obs}}{R_{rest}+R_{obs}}$$

where $R_{equ}$ is the equivalent radius of curvature of the patch.

As the resting tension is a characteristic parameter of any patch clamp system, it can be formulated in the form of the Laplace equation:

(9) $$T_{rest}=\frac{P_{0}R_{rest}}{2}$$

where $R_{rest}$ is the radius of curvature of the patch in the initial state (it is thus of little consequence which direction of curvature is present at rest) and $P_{0}$ is the equivalent pressure required for flattening of the patch in the pipette. In a similar manner, from the Laplace equation, the tension produced by the applied pipette pressure, *P*, is found to be:

(10) $$T_{Lap}=\frac{PR_{equ}}{2}$$

Now, with these modifications, all states of the patch in the pipette (including the transition state) can be explained by Laplace’s law as follows;

(11) $$T_{total}=\frac{R_{rest}}{2}(P_{0}+\frac{R_{obs}\;}{R_{rest}+R_{obs}}P)$$

**Figure 6:**
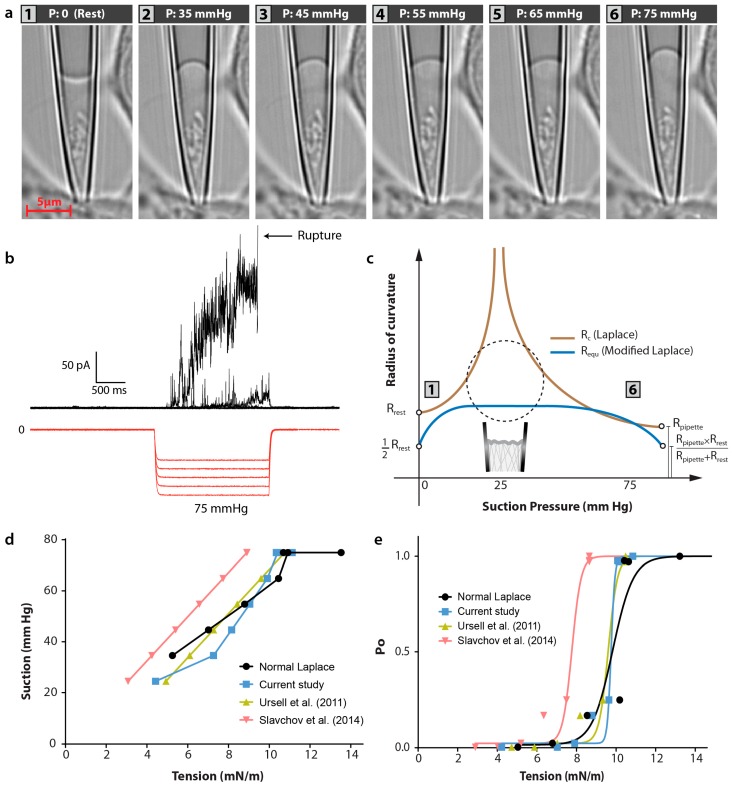
High-resolution images of a cell-attached patch containing G22S-MscL-cGFP channels and corresponding current activated during steps of negative pressure. (**a**) Images of a cell-attached membrane patch from a HEK293T cell; (**b**) Activation of G22S-MscL-cGFP channels by increasing 2s pressure steps from −25 to −75 mmHg (ΔV_patch_ = +5 mV); (**c**) Comparison between radius of curvature, R_c_, of Laplace equation and equivalent radius of curvature, R_equ_, used in our modified Laplace’s equation. This R_equ_ is more adequate for estimating the tension distribution in semi-flat states of the membrane patch as well as other patch clamping situations; (**d**) Calculation of stress induced by suction in all 6 stages of patch clamping using different techniques [42,43] (**e**). Open probability of G22S-MscL-cGFP channels from record (**b**) plotted against the tension calculated using different methods [42,43].

In this explanation, using equivalent radius and pre-stress terms, the new modified form of Laplace equation will be more adequate for estimating the membrane tension distribution in all patch clamping situations. For example, using the new formulation, the challenge of radius and stress infinity are clarified and resolved (Figure 6). In addition we have now introduced resting pre-stress such that the adhesion energy is implicit in our calculations. 

As shown in Figure 6d, the calculation of membrane tension during a patch-clamp experiment using our modified equation closely matches the results using a model proposed by Philips and coworkers [42] , who used a linear elastic model to explain how glass-bilayer adhesion affects electrophysiological experiments on mechanosensitive channels due to the effect of the glass-bilayer adhesion on mechanical properties of a bilayer patch. Here we use their average calculated adhesion tension of 2 mN/m and as a result both our and their model give very comparable results for calculated membrane tension (Figure 6d). As mentioned, one difference here is that our model has the pre-stress value implicitly within it. Another difference between their and our model is that Philips and coworkers’ model predicts a linear relationship between membrane tension and applied suction while our model predicts saturation of stress near the rupture region. In contrast, there is a significant discrepancy between our calculations of membrane tension (and those by Ursell *et al.*, [42]) and calculations based on adhesion tensions taken from Slavchov *et al.*, [43], which for the same range of negative pressures gives lower values for membrane tension of ~4–6 mN/m. This reason is simply due to the lower pre-stress value calculated by Slavchov *et al.*, which we should point out is relevant for azolectin and was not calculated for a biological membrane such that we have used in our experiments [43]. The value from Slavchov *et al.* is ~0.2 mN/m, which is an order of magnitude lower than the average adhesion energy of ~2 mN/m determined by Philips and colleagues [42], who also showed that the resting tension of liposome patches largely varied with the type of lipids of which bilayers were made (0.4–4 mN/m). Thus, here we show that by introducing an implicit term to take account for this adhesion energy we can accurately assess this not only between patches but also within the same patch over time simply by measuring the pressure (P_0_) required to overcome the resting patch radius (R_rest_).

**Figure 7:**
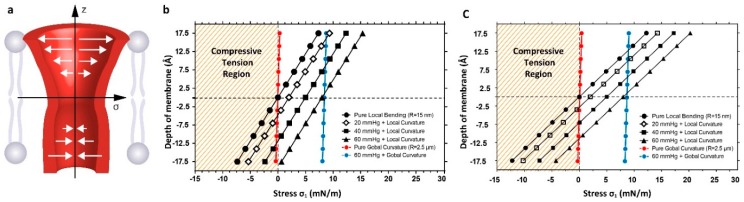
Stress distribution along the membrane thickness in response to outward bending (outward bending). (**a**) Schematic stress distribution along the channel in the lipid bilayer showing two distinct regions, which experience compressive and tensile stress; (**b**) Normal stress ($\sigma_{x}$) distribution due to the local curvature and superposition of suction pressure of 20, 40 and 60 mmHg for a radius of curvature of R_L_ = 25 nm and (**c**) radius of curvature R_L_ = 15 nm. The red line in both illustrates the stress distribution in the presence of global curvature R_G_ = 2.5 μm alone while the blue line represents global curvature superimposed on 60 mmHg applied pressure. The pressure profile is the opposite under inward bending.

Our equivalent patch model (described in the methods) enabled us to capture the phenomenological details of the lipid bilayer deformation during suction and bending of the membrane. Obviously, here we only assume the membrane as a bilayer as opposed to a biological membrane studded with proteins and linked to the cytoskeletal scaffold. We simplified the pressure profile of the lipid bilayer with different acyl chain lengths in a mean field manner and assigned it to each of the three main sections in our model (see Bavi *et al.*, 2014, for the details). We then applied local bending in the range of physiologically relevant curvatures (R_L_ ≈ 15–25 nm) [44,45]. We considered the stress distribution through the bilayer at the activation threshold of three MS channels: (i) Piezo ≈ 2 mN/m, (ii) MscS ≈ 5 mN/m and (iii) MscL ≈ 8mN/m. These membrane tensions are equivalent to negative pressures of ≈20, ≈40 and ≈60 mmHg, respectively, in our model using a pipette radius of 2 μm [5]. We then investigated what happened to the distribution of stress in regions of local curvature by looking at a superposition of the stress resulting from both local curvature and pipette suction (Figure 7). Of course, in biological environments there is likely to be plasticity in these regions of high local curvature. In some instances the regions will flatten easily in response to a threshold load and in others structural proteins will resist the loss of local curvature. Here, our interest is in deciphering how the stress distribution changes as the local curvature regimen changes, *i.e.*, as a caveolae flattens, in the presence of a force applied perpendicular to the membrane.

**Figure 8:**
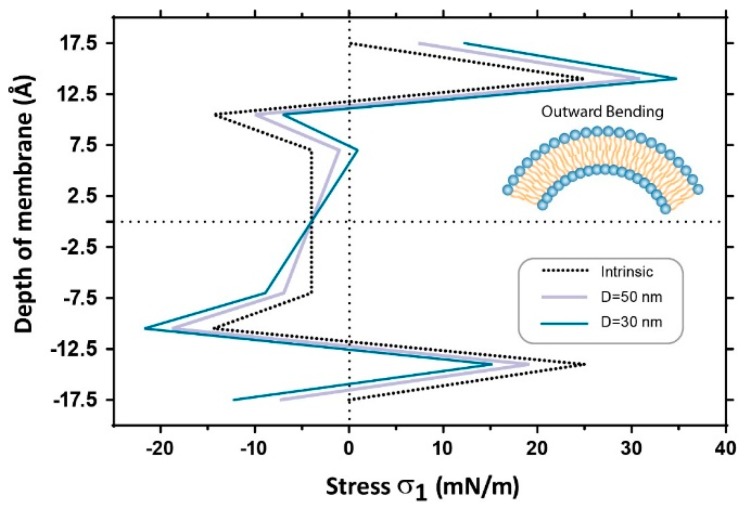
Change in the pressure profile along the membrane thickness due to the application of two levels of local curvature (Outward bending). This is equivalent to the curvature seen upon PLC activation, which cleaves PIP2 in the inner bilayer leaflet to produce diacylglycerol (DAG), which in turn can change local curvature of the membrane. This curvature also mimics the addition of lysophosphatidylcholine (LPC) or a similar shaped lipid into the outer leaflet. The opposite is true for negative curvature in this situation.

If we combine this analysis with experimental data looking at incorporation of amphipathic compounds or conical lipids into membranes it provides further credence to the effects we are seeing [3,4,7,37]. We can then estimate the amount of force generated in these local curvature regimens. For example, using physiologically relevant degrees of curvature (25–150 nm) [33,44,45], and then applying a pressure on the patch we change both the local and global curvature (even smoothing this local curvature [40]), one may calculate the local curvature as below:

(12-a) $$M_{1}=D\;(\chi_{1}+\nu \chi_{2})$$

(12-b) $$M_{2}=D\;(\chi_{2}+\nu \chi_{1})$$

where D is the Flexural stiffness of the patch which can be calculated as $D=Eh^{3}/(12(1-\nu^{2}))$ [25].

If the values of the thickness (h), elastic modulus (E), and Poisson's ratio (ν) of the membrane are assumed to be 35 Ǻ, 15 MPa and 0.5, respectively, the moment created by the curvature change of 0.06 nm^−1^ is about 12.3 pN, which is in the range of moments previously reported to initiate structural changes in mechanosensitive ion channels (14.6 pN to rotate TM1 of MscL by 10 degrees) [26].

## 4. Discussion

Here we have investigated the influence of local curvature (r < 100 nm) on MS channels and introduced an energetic term to describe its impact. As previous studies have concluded, we show that global curvature (diameter ~5 μm) is of negligible energetic consequence for a channel (~5 nm) in a patch. In an attempt to make this as obvious as possible and in no way facetious, this is similar to the curvature experienced by an ant (0.2 cm) walking on a weather balloon (2.4 m). In the case of reconstituted channels in pure lipid bilayers we have shown this exact fact using the mechanosensitive channel MscS. When calculating membrane tension, there is no difference in channel gating when positive (inward global curvature) as opposed to negative (outward global curvature) pressure is applied [46]. It is important to note that when pressure is considered in this situation though there is a difference such that the positive pressure required to gate the channel is approximately 20% higher than the negative pressure required. However, this is simply due to the radius of the global curvature of the patch and can easily be explained using a simple form of Laplace’s law [46]. 

The contribution of a change in the membrane curvature for the overall membrane tension is analytically shown by Equations (2) and (3). In Equation (3), the first term, ε, describes the contribution of membrane tension caused by, for example, negative pressure (suction) applied to the patch area in a patch clamp set up or due to an osmotic gradient imposed onto the membrane. The contribution of a change in the local curvature χ is increased by a factor of bilayer thickness, zχ. This has two meanings, one that only local curvatures (radius of curvature < 100 nm) will result in χ values that can significantly change the overall membrane stress/strain, and the other is the contribution of curvature varies along the membrane thickness with the lowest impact in the membrane mid-plane and the highest impact close to the lipid heads. The later one is specifically important with regards to the positioning of the channel gate in the bilayer as the zχ value can be either positive (tensile) or negative (compressive).

Our FE simulations document this stress variation through the thickness of the bilayer in response to the application of local curvature and pipette suction on the model membrane in a simulated patch clamp experiment. Our Finite element model suggests that in regions of high local curvature R_L_ = 25 nm, which is in the range of experimentally determined values for caveolae and other structures, the pressure profile changes by up to 20%. Previous reports suggest that changes of up to 10% can lead to MS channel activation [26]. Thus it seems plausible that bending at this local level can provide sufficient force to gate a mechanosensitive channel or conversely prevent it from opening. Furthermore, the localization of channels in regions of differing local curvature may explain some reports of channels being differentially sensitive to positive and negative pressure applied to a patch pipette. In this study we introduced a term, which takes into account the change in curvature during force application to probe this point. We found that when we applied force to these regions of high local curvature that there were striking asymmetric distributions of stress along the z-plane of the channel. Furthermore, the more sensitive to tension the channel is the more important these asymmetric stress distributions become. 

Energetically speaking our computational results fit well together with previous experimental results. However, it is clear that the response of a channel to this local curvature is intimately linked to its three dimensional structure. This includes whether the channel itself can induce local curvature in the bilayer. For example, recent work clearly shows that MscS-like channels produce a degree of inward bending around themselves deforming their surrounding lipids [20,47]. Thus, the effect of local curvature on members of the MscS-like channel family is of great interest and may well modify the stress felt by this family of MS channels [48,49,50]. Indeed, certain members of this diverse family may in fact have their own curvature inducing domains such as the *E. coli* MscS paralogue YjeP [50,51]. 

**Figure 9:**
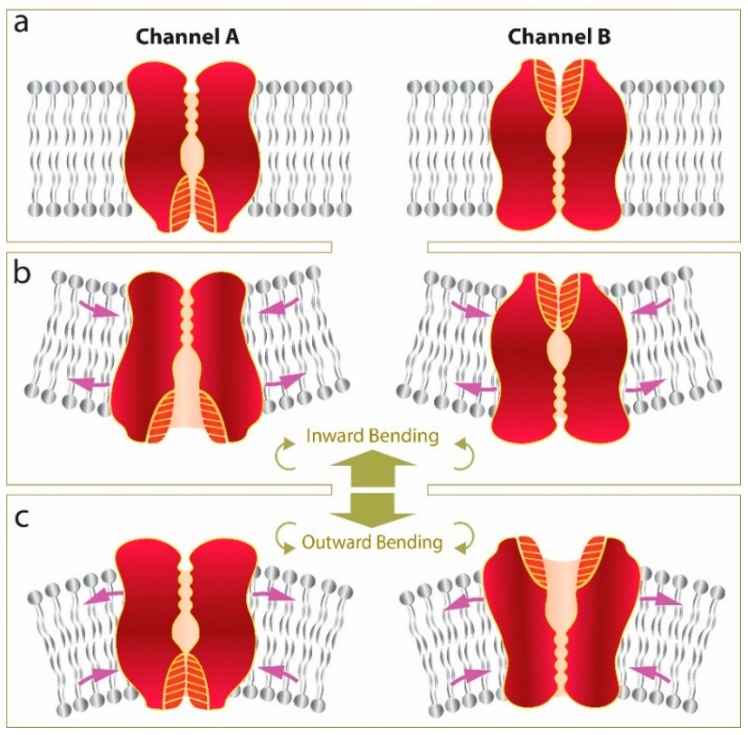
Schematic representation of how local bending can have different effects on gating of structurally different channels (Channel A and Channel B). If we assume both channels are in the closed state (**a**); bending of the membrane in the inward direction (**b**); tends to open Channel A but close Channel B. Conversely, applying local bending in the outward direction (**c**) tends to close Channel A and open Channel B.

As mentioned previously, in addition to the strength of interactions of the channel with the bilayer the position of its gate is also of critical importance. Thus, if we take two fictional channels of identical structure differing only by the position of their gate, one being intracellular (Channel A) the other being at the extracellular side (Channel B), it becomes clear that the asymmetric stress distribution that we see in our FE model generated by local curvature can have opposite effects on the two channels (Figure 9). In this scenario bending of the membrane in the inward direction (Figure 9b) is favorable for channel A opening but not for channel B and *vice versa* for activation of the channels in response to outward bending. 

Thus it becomes easy to imagine a scenario where channel structure determines the sensitivity to different curvature regimens (inward and outward). This would endow a cell with an even larger repertoire of parameters to fine-tune the response of MS channels and focus mechanical forces [52] regardless of their gating mechanism (force-from-lipids or force-from-filament) ([12,14]). 

Interestingly, we can take this scenario (Figure 9) and begin to apply it to the MscL channel family. For example, in contrast to MscL of *E. coli* (EcMscL) its homologs from *Mycobacterium tuberculosis* (TbMscL) and *Lactococcus lactis* (LlMscL), which require almost lytic tension for their activation, can be activated with much lower LPC concentrations compared to EcMscL, whose activation by LPC was directly proportional to its sensitivity to membrane tension [53,54] These results suggest that channels, high in sequence similarity, can have differential sensitivity to LPC and activation by membrane tension. However, whether this is due to the local curvature induced by LPC or other effects such as changes in line tension are at this point unknown. Given our data and how local curvature can affect stress distribution through the bilayer thickness, it really begs the question how can homologues with such high sequence similarity be so different in their response? In depth functional and structural analysis of these homologues will likely offer insights into how this differential sensitivity is generated and may provide us with structural clues relevant to other eukaryotic channels. Potentially these homologues are more sensitive to LPC as they are for some reason more sensitive to local curvature. Regardless of the answer given the pervasive nature of membrane curvature it is hard to imagine that local curvature is not a relevant physiological stimulus for mechanosensitive channels.

## 5. Conclusions

Most importantly and unequivocally, we show that the global curvature of a patch, which is visible under the microscope during patch clamp experiments, is of negligible energetic consequence for activation of an MS channel in a model membrane. The local curvature (R_L_ < 50 nm) encountered in biological membranes such as caveolae not only can produce forces local to a channel in the order of those generated by applying pressure in the pipette but they can also vastly modify the forces felt by a channel localized to this region during force application. While no channels have yet been robustly proven to localize to regions such as caveolae, there are many other regions in the cell that have high local curvature. Thus, it is of critical importance to understand the energetic impact of differing length scale curvature regimens on mechanosensitive channels. 
